# *Colletotrichum tofieldiae* enhances phosphorus uptake and biomass production and alters the microbial interactions in the rhizosphere of komatsuna (*Brassica rapa* var. *perviridis*) grown in phosphorus-deficient farm soils

**DOI:** 10.5511/plantbiotechnology.25.0529a

**Published:** 2025-09-25

**Authors:** Elsie Sarkodee-Addo, Yasuhiro Tsujimoto, Aung Zaw Oo, Tomohiro Nishigaki, Kei Hiruma, Papa Saliou Sarr

**Affiliations:** 1 Crop, Livestock and Environment Division, Japan International Research Center for Agricultural Sciences, 1-1 Ohwashi, Tsukuba, Ibaraki 305-8686, Japan; 2 Plant Microbiology Laboratory, Tokyo University of Agriculture and Technology, 3-8-1 Harumicho, Fuchu, Tokyo 183-8538, Japan; 3 Department of Life Sciences, Graduate School of Arts and Sciences, The University of Tokyo, 3-8-1 Komaba, Meguro-ku, Tokyo 153-8902, Japan

**Keywords:** bioinoculant, Brassicaceae, *Colletotrichum tofieldiae*, phosphorus-deficient soil, plant growth

## Abstract

Microbial bioinoculants should play important roles to achieve sustainable phosphorus (P) management in agriculture. *Colletotrichum tofieldiae* (Ct), a fungal endophyte, is a promising material that supports phosphorus acquisition in *Arabidopsis* under low phosphorus (P) conditions and promotes maize and tomato growth in a greenhouse. However, its effects on leafy vegetables such as komatsuna, particularly those with a range of P availability in the soil, remain largely unclear. This study evaluated the impact of Ct inoculation on komatsuna growth, P uptake, rhizosphere soil and plant microbiological properties in sterilized and non-sterilized farm soils in Japan. The Ct mycelium was incubated in a mixture of rice bran, wheat bran, and wood chips for two weeks, and then applied to the soils at 0%, 1%, and 5% mass ratios (Ct0, Ct1, Ct5). Ct0 was the sterilized medium applied at a 5% mass ratio. Non-sterilized and sterilized soils received 20 and 80 mg P kg^−1^ as low and high P treatments, respectively. Ct significantly increased plant biomass in a manner dependent on the initial inoculation dose in Low P- and High P-amended soils, although Ct-mediated plant growth promotion was more prominent in the low P-supplemented soils. Furthermore, Ct inoculation was found to increase microbial activity such as P solubilization, in rhizosphere soil and/or roots under both P conditions. This study is the first to demonstrate the positive influence of Ct on Brassicaceae growth in not only P-deficient but also P-supplemented soils, confirming its potential to improve plant development under P-deficient and P-supplemented soil conditions.

## Introduction

Sustainable agriculture is gaining momentum because of the need to reduce the dependence on chemical fertilizers while maintaining optimal plant growth and productivity (Hardoim et al. 2015). The excessive and prolonged use of synthetic fertilizers has led to environmental degradation, including soil acidification, reduced microbial diversity, and nutrient imbalances (Fierer 2017; Savci 2012). Additionally, the rising costs of chemical fertilizers, exacerbated by supply chain disruptions and geopolitical factors, threaten food security, particularly in phosphorus-deficient regions (Cordell et al. 2009). In this context, biological alternatives, such as microbial biofertilizers, offer promising solutions for improving soil fertility and plant growth while minimizing environmental harm.

Endophytes, including *Colletotrichum tofieldiae* (Ct), have emerged as valuable biological agents capable of establishing symbiotic relationships with plants, increasing nutrient uptake, and stress tolerance (Hiruma et al. 2016; Rodriguez et al. 2009). These microorganisms facilitate plant growth through multiple mechanisms, including organic acid production, phosphorus solubilization, and the stimulation of plant hormone synthesis (Berg et al. 2014; Smith and Read 2008). Phosphorus (P), an essential macronutrient for plant growth, plays a crucial role in energy transfer, photosynthesis, and nucleic acid synthesis (Raghothama 1999). However, its bioavailability in soil is often limited because of its tendency to form insoluble complexes with iron, aluminum, or calcium, depending on the pH (Hinsinger 2001; Holford 1997). This limitation reduces crop productivity and necessitates high fertilizer inputs, which are both costly and environmentally detrimental. To overcome phosphorus deficiency, plants have evolved several adaptive strategies, including changes in root architecture, increased secretion of phosphatases, and the establishment of mutualistic interactions with beneficial microbes (Richardson and Simpson 2011) including bacteria and fungi. For instance, efficient phosphorus cycling in soil is largely mediated by microbial processes that mobilize both inorganic and organic forms of P. The *gcd* gene more common in bacteria, encodes glucose dehydrogenase, a major enzyme responsible for solubilizing inorganic phosphate through organic acid production (Chen et al. 2016), while *pqqE* is involved in the biosynthesis of pyrroloquinoline quinone (PQQ), an essential cofactor that enhances *gcd* enzymatic activity (Choi et al. 2008). In addition, the bacterial *phoD* gene encodes alkaline phosphatase, an enzyme critical for the mineralization of organic P compounds (Bergkemper et al. 2016). Therefore, monitoring the abundance of the *gcd*, *pqqE*, and *phoD* genes provides an integrated view of the microbial mechanisms contributing to soil P availability. While P-solubilizing bacteria play a crucial role in P mobilization, fungal endophytes such as Ct have demonstrated the ability to improve plant fitness under low-P conditions by increasing phosphorus uptake and alleviating nutrient stress (Díaz-González et al. 2020; Hiruma et al. 2023).

Komatsuna (*Brassica rapa* var. *perviridis*), a leafy vegetable widely cultivated in East Asia, is highly valued for its rich nutritional content and adaptability to diverse growing conditions (Singla et al. 2013). However, its phosphorus demand for optimal growth and yield is substantial, making it particularly susceptible to phosphorus limitation (Mar et al. 2012). While Ct has been shown to promote plant growth under low-P conditions in various plants, including *Arabidopsis thaliana* (Hiruma et al. 2016), maize and tomato (Díaz-González et al. 2020), its specific effects on komatsuna grown in natural soil conditions, as well as its interactions with soil microbial communities remain unexplored. Liu et al. (2021) reported that inoculation of wheat with *Fusarium pseudograminearum* enriched the plant-associated microbiome, which associated with improved plant growth and enhanced disease resistance. Building on these findings, the present study aims to clarify whether Ct inoculation could similarly exert a positive influence on the overall soil microbial community. Expanding the understanding of the role of Ct across diverse plant systems, particularly in crops with high phosphorus demand, such as komatsuna, could offer valuable insights into sustainable agricultural practices.

This study aimed to investigate the potential of Ct as a biofertilizer for komatsuna by evaluating its effects on plant physiological responses, phosphorus uptake, and microbial community dynamics under different soil phosphorus conditions. Our findings could provide valuable insights into the application of Ct as a sustainable alternative to chemical fertilizers, improving crop productivity while supporting soil health and resilience in the face of global agricultural challenges.

## Materials and methods

### Authentication test for komatsuna root infection by Ct

A pot experiment was conducted to visualize Ct colonization in plant tissues after inoculation. Komatsuna seeds were surface-sterilized by sequentially treating them with 5% sodium hypochlorite (NaClO) solution for 10 min, followed by 75% ethanol for 3 min. The sterilized seeds were then rinsed five times with sterile distilled water and sown in 500-ml pots filled with autoclaved vermiculite (120°C, 20 min) and half-strength Hoagland solution. Two treatments were applied: with and without Ct inoculation, each replicated three times. For the Ct inoculation treatment, Ct was initially grown on Petri dishes containing 2% PDA, supplemented with streptomycin and other antibiotics to prevent bacterial growth, for 2 weeks to allow mycelial development. A small portion of the mycelium was then cut from the agar plate and placed near the sown komatsuna seeds. The pots were covered with aluminum foil and incubated at 25°C for 4 days, after which the aluminum foil was removed. The plants were grown for another 7 days, at which point the plants were sampled by carefully removing the roots and placing them, along with the stems, in a 50-ml Falcon tube containing 50% formaldehyde for subsequent microscopic observation. The root system, junctions between roots and stems, and stems were examined under a modular upright compound microscope Olympus BX43 (Olympus Corporation, Tokyo, Japan) to detect the presence or absence of Ct. For imaging, the microscope was equipped with an Olympus DP21 digital camera, and images were captured using Colleen’s Standard software. The microscope was configured for bright-field observation, using a UPLFLN plan fluorite objective lens at magnification of 100 x.

### Preparation of *Colletotrichum tofieldiae* media as inoculum

A substrate mixture was prepared by combining commercially available grinder wood chips, rice bran, and wheat bran at a mass ratio of 3 : 1 : 1 (180 : 60 : 60 g). Distilled water (approximately 120 ml) was added, and the mixture was thoroughly mixed with the substrate. The mixture was then transferred to a conical flask and autoclaved at 120°C for 20 min to ensure sterility and eliminate potential contaminants. After cooling, the sterilized substrate was inoculated with *Colletotrichum tofieldiae* by introducing its mycelium, which had been subsequently grown on a potato dextrose agar (PDA) plate for two weeks at 25°C in the dark. Before the mycelia were cultured, the PDA was autoclaved at 120°C for 20 min, and after cooling, the antibiotics streptomycin (125 mg l^−1^) and rifampicin (100 mg l^−1^) were added to prevent bacterial growth in the medium. A small portion of the Ct inoculum was placed at the center of the PDA plate, which was then covered with aluminum foil to maintain darkness and incubated at 25°C for approximately two weeks to allow mycelial development. Once fully grown, the Ct mycelium was transferred into the prepared substrate flask and evenly dispersed by thorough mixing. The flask was then covered with aluminum foil and incubated in a dark chamber at 25°C for 14 days. Regular monitoring was conducted throughout the incubation period to assess fungal growth and maintain appropriate humidity under carefully sterilized conditions.

### Soil for pot experimentation

The soil used in this experiment was upland soil collected from the JIRCAS experimental farm in Tsukuba, Japan. It is classified as an Andosol, characterized by high P fixation (99%) and low P availability of 7 mg kg^−1^ (Bray II), as reported by Oo et al. (2020) and Dinh et al. (2023). Before inoculation, the soil was air-dried, sifted through an 8-mm sieve, and divided into two portions. One portion was sterilized through two series of consecutive autoclaving cycles at 120°C for 20 min to minimize the influence of soil borne microbial communities on the experiment. The second portion remained untreated, preserving its natural microbial community. This non-sterilized condition aimed to replicate typical agroecosystem soil conditions, enabling comparisons with sterilized soil. The pots were subsequently filled with 600 g of air-dried soil.

### Preparation of soil nutrients and *Colletotrichum tofieldiae* inoculation

Inorganic fertilizer was formulated using NH_4_/NO_3_, K_2_SO_4_, and NaH_2_PO_4_-2H_2_O as sources of nitrogen (N), potassium (K), and phosphorus (P), respectively. N and K were applied at a rate of 200 mg per kg of air-dried soil (hereafter, mg kg^−1^), ensuring an equal dosage for both elements. Phosphorus was supplied at two levels: a low rate of 20 mg kg^−1^ (Low P hereafter) and a high rate of 80 mg kg^−1^ (High P hereafter). These P levels were selected on the basis of a preliminary test in which soil, supplemented with the same N and K application rates, was fertilized with 0, 6, 20, or 80 mg P kg^−1^ and used to grow komatsuna for 26 days. The plants grown with 0 and 6 mg P kg^−1^ remained very small, whereas those grown with 20 mg P kg^−1^ presented some development but limited growth. In contrast, plants receiving 80 mg P kg^−1^ exhibited robust growth (Supplementary Figure S1). Based on these observations, 20 mg P kg^−1^ and 80 mg P kg^−1^ were chosen to represent Low P- and High P application conditions, respectively. The prepared Ct substrate was added to the pots at three different concentrations: 0, 1, and 5% (hereafter referred to as Ct0, Ct1, and Ct5, respectively). The Ct0 treatment was prepared by thoroughly mixing 95 g of soil with 5 g of autoclaved Ct substrate to make a 100 g soil mixture. The Ct1 treatment was prepared by mixing 1 g of non-autoclaved Ct substrate with 4 g of autoclaved Ct substrate and then combining it with 95 g of soil. For the Ct5 treatment, 5 g of non-autoclaved Ct substrate was mixed with 95 g of soil to reach a total of 100 g per pot. Since the pots received 600 g of soil mixture, these fractions were added six times each. The soil and Ct substrate were added on a dry weight basis.

### Experimental design

Komatsuna seeds were sterilized as described above. After rinsing five times with sterile distilled water, the seeds were directly sown into 1-kg plastic pots containing a mixture of soil and Ct substrate (600 g).

The experiment followed a completely randomized design with five replications. The treatments included factorial combinations of two P application rates (Low P and High P), two soil conditions (sterilized by autoclaving and non-sterilized), and three Ct concentrations Ct0, Ct1, and Ct5. In total, 60 pots of komatsuna plants were cultivated under controlled conditions of 25°C/20°C day/night temperatures for 32 days. The soil moisture was adjusted to 35% (weight basis) throughout the experiment by periodic watering to ensure adequate water availability using sterilized distilled water.

### Measurement of plant growth parameters and determination of plant phosphorus content

The measurement of plant growth parameters began ten days after Ct inoculation and continued weekly until the plants reached their maximum vegetative stage at 32 days. Key growth parameters, including leaf length and leaf number, were recorded at 10-, 17-, 24-, and 32 days post-inoculation (dpi) to assess the impact of Ct on plant development and overall growth trajectory.

Komatsuna plants were harvested 32 dpi to determine the total biomass as the sum of shoot dry weight and root dry weight. Before drying, leaf, stem, and root samples were collected and rinsed with 70% ethanol for sterilization, and approximately 100 mg of subsamples were placed into 2-ml tubes containing two sterile 5-mm zirconium balls. These samples were flash-frozen in liquid nitrogen and then pulverized using a Master Shaker machine at 1000 rpm for 1 min. The powdered samples were stored at −20°C until DNA extraction for microbial abundance quantification in plant tissues. A second subsample from each plant was used to determine the moisture content of the fresh biomass.

The P concentrations in the shoot and root samples were analyzed using the molybdenum blue method (Murphy and Riley 1962). Approximately 0.2 g of dried, crushed plant biomass was transferred into a 50-ml digestion tube. An 8-ml mixture of concentrated CHNO_3_ : CHClO_4_ (3 : 1 ratio) was added, and the tube was placed in a heating block overnight. Digestion was performed at 105°C for 60 min, followed by CHNO_3_ removal at 140°C for 60 min. Silicate dehydration was then carried out at 170°C for approximately 35 min. After cooling, the digested mixture distillate was obtained and titrated with H_2_SO_4_. A 0.5 N NaHCO_3_ buffer was used for initial phosphorus extraction, followed by ascorbic acid treatment to extract phosphorus from the plant tissues. The resulting blue color intensity was measured at 380 nm, and P concentration was calculated using a standard curve. The shoot and root P contents were calculated as the product of the dry weight and P concentration of each part. The total P uptake was calculated as the sum of the shoot and root P contents.

### Soil sampling at harvest

Soil sampling was conducted on the harvest day, corresponding to 32 dpi. The plants were carefully removed from the pots, and the loose soil was gently shaken. The rhizosphere soil—defined as the soil still attached to the roots after shaking—was carefully removed using clean brushes. These rhizosphere soil samples were placed in Ziplock bags and stored at −20°C until DNA extraction and subsequent molecular analysis.

### DNA extraction from rhizosphere soil and plant tissues, and microbial gene quantification

DNA was extracted from 0.4 g of thawed rhizosphere soil from the −20°C storage using the Fast DNA Spin Kit for Soil (Mo-Bio, Carlsbad, CA, USA), with modifications as described by Sarr et al. (2020a). The soil moisture content was determined concurrently during the extraction. DNA from the plant roots and shoots was extracted using the Plant Mini Kit, according to the manufacturer’s instructions.

The extracted DNA was used to quantify the abundance of several microbial genes in both the soil and plant tissues through real-time PCR. These target genes included *16S rRNA* for total bacteria, *ITS* for total fungi, Ct tubulin 2 sequence (CT04_11973) for the *Colletotrichum* fungus (Hiruma et al. 2016), *gcd* involved in inorganic phosphorus solubilization, *pqqE* (pyroquinoline quinone gene) which is a co-factor of *gcd*, the alkaline phosphatase *phoD*. In the rhizosphere soil, we quantified the abundance of *16S rRNA*, *ITS*, Ct, *gcd*, *pqqE*, and *phoD*, whereas in plant roots, stems, and leaves, *16S rRNA* and *ITS* were assessed. The sequence information of primers and the annealing temperatures are provided in Supplementary Table S1.

Prior to qPCR, rhizosphere soil DNA samples were diluted 10 times with pure water to reduce possible inhibitors. The plant DNA was amplified without dilution since inhibitors are less prevalent in plant tissues. For each target gene, standard plasmids were prepared as described in Sarr et al. (2020b) and Sagnon et al. (2022). The detailed qPCR conditions followed Sagnon et al. (2022), and the amplification efficiency/r^2^ values were in the ranges of [89–101%] and [0.996–0.999], respectively. The starting quantities generated from qPCR were used to calculate the final microbial gene abundance (copy number g^−1^ dry soil), considering the dilution factor, the amount of soil used for extraction, and the soil moisture content (Sarr et al. 2020a).

### Statistical analysis

Statistical analyses were conducted using JMP v14.0.0 software (SAS Institute Inc., Tokyo, Japan). Normality and homogeneity of variance were checked using the Shapiro–Wilk and Levene tests, respectively. Certain variables were log-transformed to ensure normal distribution. After outliers were removed via the Smirnov–Grubbs test, a three-way analysis of variance (ANOVA) was conducted to assess the main and interactive effects of the soil type (S), P rate (P), and Ct concentration (Ct) on the measured variables. Replicates were treated as random effects for each experimental field. Post-hoc comparisons of means were conducted using Tukey’s honestly significant difference (HSD) test, with the significance set at *p*<0.05.

## Results

### Visualization of potential Ct colonization in komatsuna plant tissues post-inoculation

**Figure 1:**
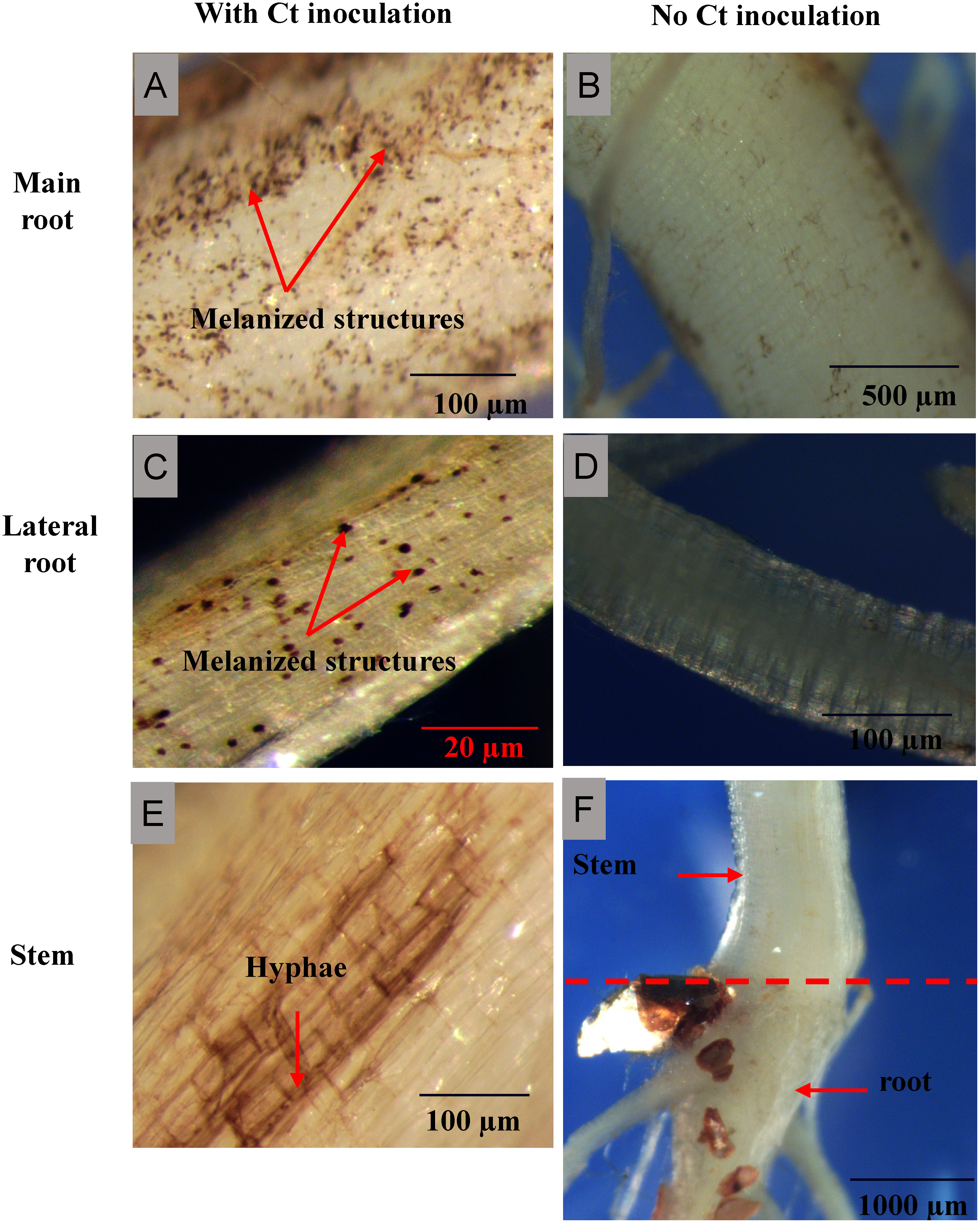
Figure 1. Visualization of Ct colonization in komatsuna plant tissues post-inoculation.

The short-term pot experiment confirmed the infection of komatsuna tissues (roots and stems) by the inoculated Ct (Figure 1). Melanized structures were observed in the main and lateral roots of komatsuna following Ct inoculation (Figure 1A, C), whereas they were absent in the roots of non-inoculated plants (Figure 1B, D). Similarly, fungal hyphae were exclusively detected in the stem tissues of inoculated plants (Figure 1E) and were absent in the stems of non-inoculated plants (Figure 1F). These findings indicate that the detected fungal structures originated from the inoculated Ct.

### Total biomass, P uptake, and temporal changes in komatsuna leaf number and length

**Figure 2:**
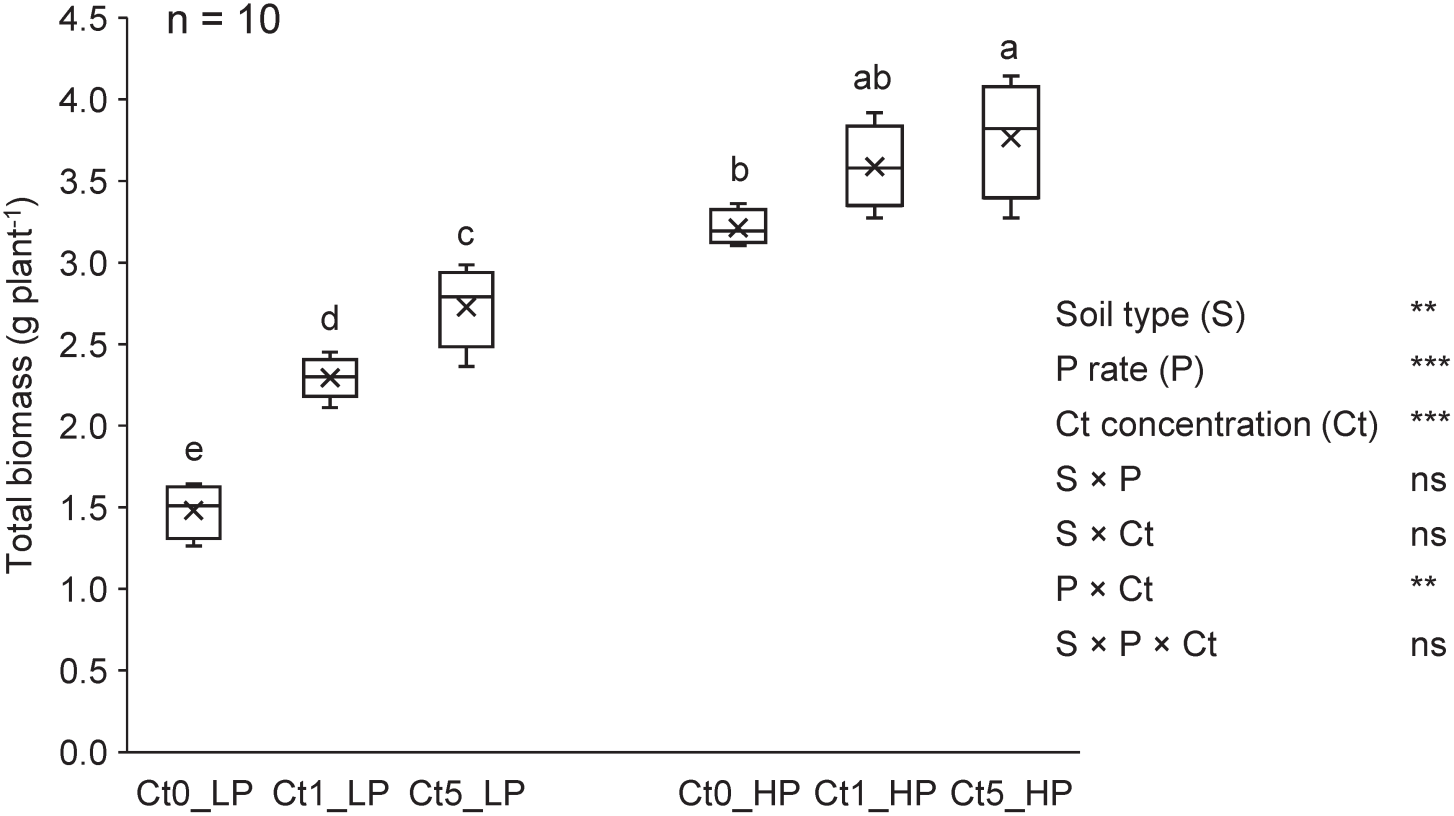
Figure 2. Total biomass of komatsuna (at 32 dpi) as affected by inoculated Ct concentration and P application rate. Different letters indicate significant mean differences at *p*<0.05 in Tukey’s HSD test. In the ANOVA summary, ** *p*<0.01, *** *p*<0.001, ns: not significant. Soil type refers to sterilized soil (SS) and non-sterilized soil (NS). P rates are 20 mg P kg^−1^ dry soil (Low P=LP) and 80 mg P kg^−1^ dry soil (High P=HP). Ct0, Ct1, and Ct5 are Ct concentrations at 0, 1, and 5% mass ratio of viable cells, dpi: days post-inoculation. *n* (top left corner)=number of biological replicates (5 replicates from SS+5 replicates from NS).

**Figure 3:**
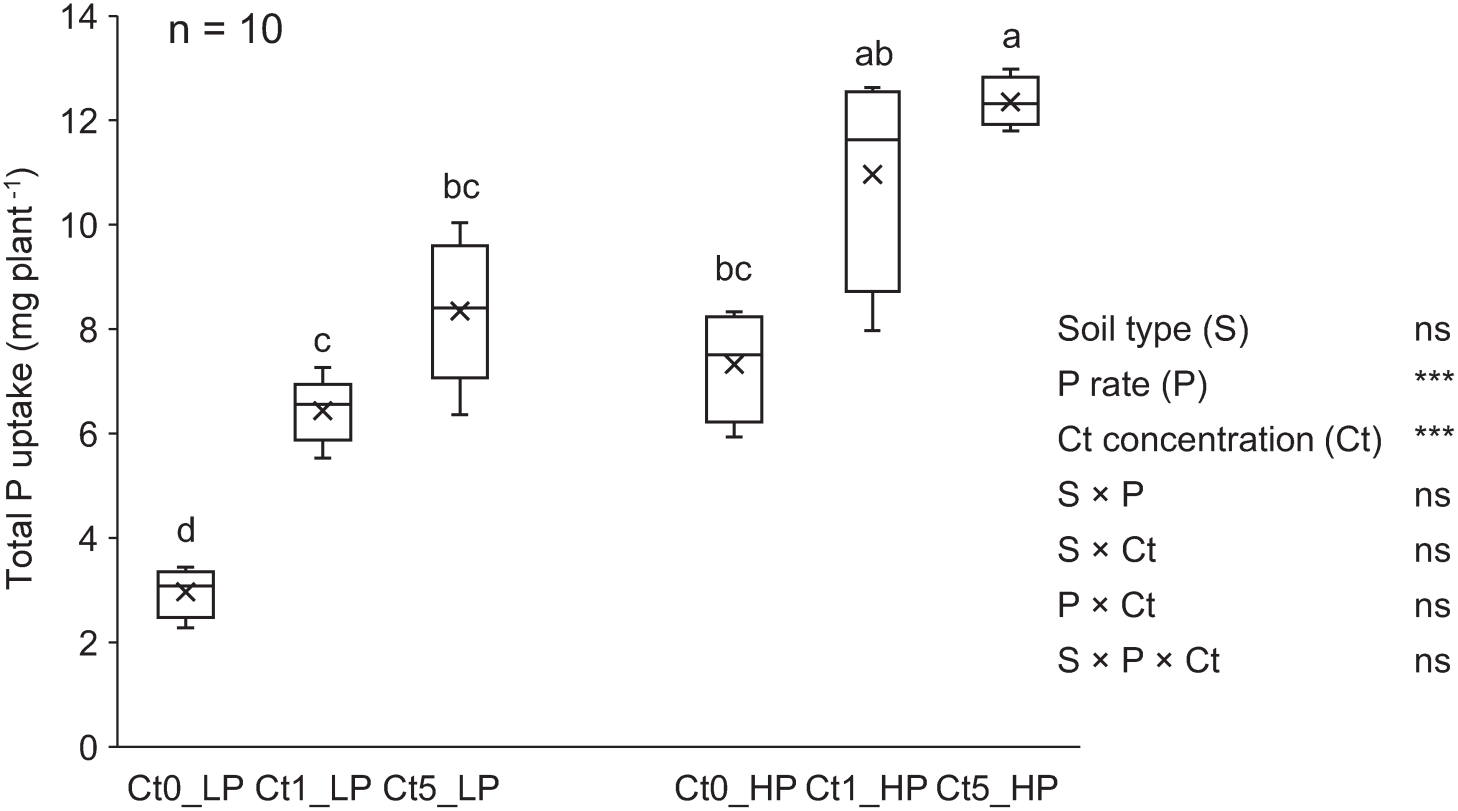
Figure 3. Total P uptake of komatsuna (at 32 dpi) as affected by inoculated Ct concentration and P application rate. Different letters indicate significant mean differences at *p*<0.05 in Tukey’s HSD test. In the ANOVA summary, *** *p*<0.001, ns: not significant. Soil type refers to sterilized soil (SS) and non-sterilized soil (NS). P rates are 20 mg P kg^−1^ dry soil (Low P=LP) and 80 mg P kg^−1^ dry soil (High P=HP). Ct0, Ct1, and Ct5 are Ct concentrations at 0, 1, and 5% mass ratio of viable cells, dpi: days post-inoculation. *n* (top left corner)=number of biological replicates (5 replicates from SS+5 replicates from NS).

Three-way ANOVA revealed no interactive effects of soil type (S) with P application rate (P), Ct concentration (Ct) or between P and Ct, except for a significant interaction between P and Ct on total biomass (Figures 2, 3). Thus, the mean values across the two soil types were compared for the effect of Ct inoculation within each P application rate. Under Low P conditions, the total biomass of komatsuna plants significantly increased by 53% with the Ct1 treatment relative to the Ct0 treatment and further increased by 19% with the Ct5 treatment relative to the Ct1 treatment (Figure 2). The effect of Ct inoculation was less significant in the High P treatment where Ct5 resulted in a significantly greater total biomass by 17% than did Ct0, but no significant mean differences were detected between Ct0 and Ct1 or between Ct1 and Ct5. Furthermore, the non-sterilized soil presented greater plant biomass than the sterilized soil under Ct0 and Ct1 treatments, regardless of the P application rate (Supplementary Figure S2). However, under Ct5 treatment, soil sterilization had no significant effect on plant biomass.

For total P uptake, there was no significant interaction effect between the P application rate and Ct concentration, and the positive and dose-dependent response to Ct inoculation was more prominent under Low P conditions than under the High P conditions (Figure 3). Compared with the Ct0 treatment, the Ct5 treatment resulted in significantly greater total P uptake, by 181% in the Low P treatment and by 74% in the High P treatment. Significant differences in total P uptake between the Ct0 and Ct1 treatments were detected only in the Low P treatment but not in the High P treatment.

For all the leaf number counting dates, only the P×Ct interaction was significant (*p*<0.05). The temporal changes in leaf number, influenced by Ct and P, are shown in Supplementary Figure S3A. At 10 dpi, the leaf number increased with increasing Ct inoculation concentration, but not with increasing P application rate. On subsequent counting dates (17, 24, and 32 dpi), both the P application rate and the Ct inoculation concentration significantly affected the leaf number, with the leaf number increasing with increasing P rates and Ct concentrations. Specifically, at 17 dpi, the effect of P was evident only under Ct0, while no difference was observed between Ct1 and Ct5. However, the influence of Ct1 and Ct5 on leaf number exceeded that of Ct0 at each P application rate. These differences observed at 17 dpi were consistent with those observed at 24 dpi. By 32 dpi, the effect of the P application rate remained consistent across the P×Ct combinations, but the leaf number significantly increased with increasing Ct inoculation concentration in the Low P soil. In High P soil, however, no significant difference was detected between Ct1 and Ct5. At 32 dpi, the difference in leaf number between Ct0 and Ct1 or Ct5 treatments was greater in Low P soil (by 0.7 or 1.3 leaves) than in the High P soil (by 0.6 or 0.9 leaves).

With respect to the leaf length (Supplementary Figure S3B), the P×Ct interaction was significant at all measurement dates (*p*<0.05), whereas the S×P interaction was significant only at 24 dpi (*p*<0.001), and the S×Ct interaction was significant at 10 dpi (*p*<0.05). At 10 dpi, leaf length increased with increasing P application rate, but only under Ct0 and Ct5. Under Low P conditions, Ct1 and Ct5 resulted in longer leaf lengths than Ct0, whereas under High P conditions, Ct0 and Ct1 had the same effect, and were significantly lower than Ct5. Leaf elongation remained consistent until 17 dpi, with treatment differences mirroring those observed at 10 dpi. At 24 dpi, Ct1 and Ct5 led to greater leaf lengths than Ct0 under Low P, whereas under High P, Ct5 had a significantly greater effect than Ct1 and Ct0, which did not differ. At 32 dpi, the effect of the P application rate on leaf length was observed only in Ct0. Compared with Ct0, only Ct5 resulted in increased. Under High P, both Ct1 and Ct5 increased leaf length.

### Rhizosphere soil microbial abundance

**Figure 4:**
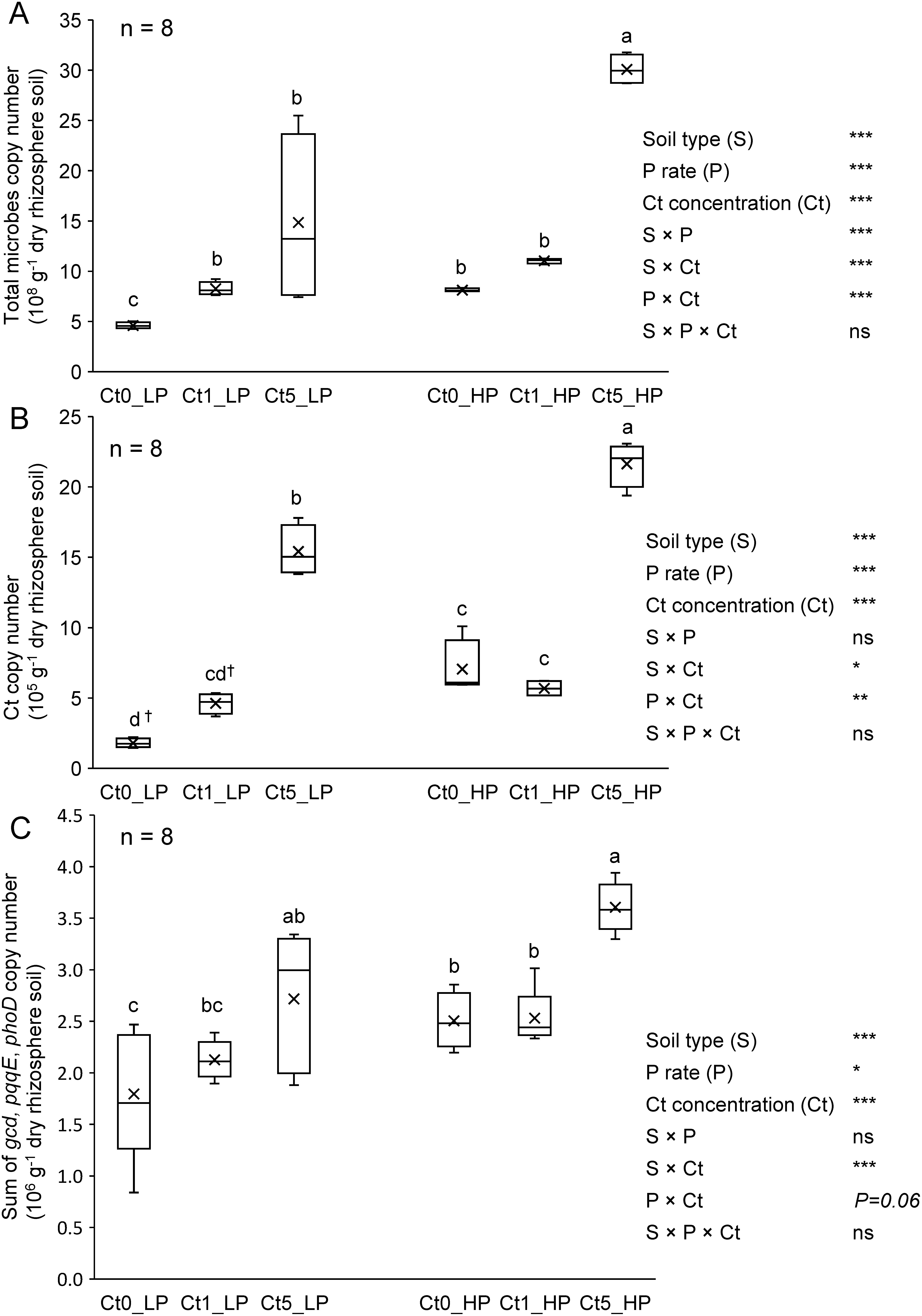
Figure 4. Abundance of total microbes (bacteria+fungi) (A), Ct fungi (B), and total of selected phosphorus-cycling microbial genes (*gcd*, *pqqE*, and *phoD*) (C) in komatsuna rhizosphere soil (at 32 dpi) as affected by inoculated Ct concentration and P application rate. For each variable, different letters indicate significant mean differences at *p*<0.05 in Tukey’s HSD test. ^†^ Increasing tendency at *p*=0.09 for the Ct abundance variable. In the ANOVA summaries, * *p*<0.05, ** *p*<0.01, *** *p*<0.001, ns: not significant. Soil type refers to sterilized soil (SS) and non-sterilized soil (NS). P rates are 20 mg P kg^−1^ dry soil (Low P=LP) and 80 mg P kg^−1^ dry soil (High P=HP). Ct0, Ct1, and Ct5 are Ct concentrations at 0, 1, and 5% mass ratio of viable cells, dpi: days post-inoculation. *n* (top left corners)=number of biological replicates (5 replicates from SS+5 replicates from NS).

The abundance of total microbes, represented by the sum of total bacteria and total fungi, Ct fungi, and the sum of selected P-cycling microbial genes in the rhizosphere soil at harvest (32 dpi), is shown in Figure 4. Based on the significance of the different interactions (S×P, S×Ct, and P×Ct) in the ANOVA summaries, we presented the abundances as influenced by the P application rate and Ct inoculation concentration. Under Low P conditions, Ct1 and Ct5 similarly increased the total microbial abundance in the rhizosphere soil, with both significantly greater than that under Ct0 (Figure 4A). However, under the High P conditions, no significant difference was observed between Ct0 and Ct1, whereas Ct5 significantly increased microbial abundance. Overall, Ct inoculation increased the abundance of soil microbes in a dose-dependent manner. Additionally, we observed an increase in soil’s microbial abundance with increasing in the P application rates. The microbial abundance was significantly higher in non-sterilized soil than in sterilized soil at each Ct inoculation concentration, with Ct5 surpassing Ct1 and Ct0 in both soils (Supplementary Figure S4). In sterilized soil, the total microbial abundance increased in a dose-dependent manner.

The Ct abundance in the rhizosphere soil increased only under Ct5, both in Low P and High P soils, regardless of the soil type (Figure 4B). Compared with Ct0, Ct1 did not significantly increase the Ct abundance in the rhizosphere soil. The Ct abundance in the rhizosphere soil was greater in the High P soil than in the Low P soil for Ct0 and Ct5; however, it remained unchanged for Ct1 (data not shown). The effect of soil type was also significant, with Ct abundance being significantly higher in non-sterilized soil than in sterilized soil at Ct0 and Ct1 inoculation concentration. However, no significant difference was observed between sterilized and non-sterilized soils at Ct5 (Supplementary Figure S5). The effect of Ct5 surpassed those of Ct1 and Ct0 in both soils, and the effect of Ct1 was significantly higher than that of Ct0 in sterilized soil.

The P-cycling microbial genes quantified included the inorganic P-solubilizing gene *gcd* and its cofactor *pqqE*, as well as the organic P-mineralizing gene alkaline phosphatase (*phoD*). The combined abundance of these genes, influenced by the P application rate and Ct inoculation concentration (P×Ct: *p*=0.06), is shown in Figure 4C. In the Low P soil, only Ct5 presented a significantly higher abundance of P-cycling genes than Ct0. In contrast, in High P soil, neither Ct1 nor Ct5 inoculation concentrations resulted in an increased abundance of P-cycling genes relative to Ct0. The P application rate had a significant effect only under Ct0, with a greater abundance of P-cycling genes in High P soil than in Low P soil. This effect was not observed under Ct1 or Ct5. Compared with sterilized soil, non-sterilized soil consistently presented a significantly higher abundance of P-cycling genes across all Ct inoculation concentrations (Supplementary Figure S6). While Ct inoculation did not significantly influence the overall abundance of P-cycling genes in non-sterilized soil, in sterilized soil, both Ct1 and Ct5 had similar effects, which were significantly greater than those of Ct0.

### Microbial abundance in plant tissues

**Figure 5:**
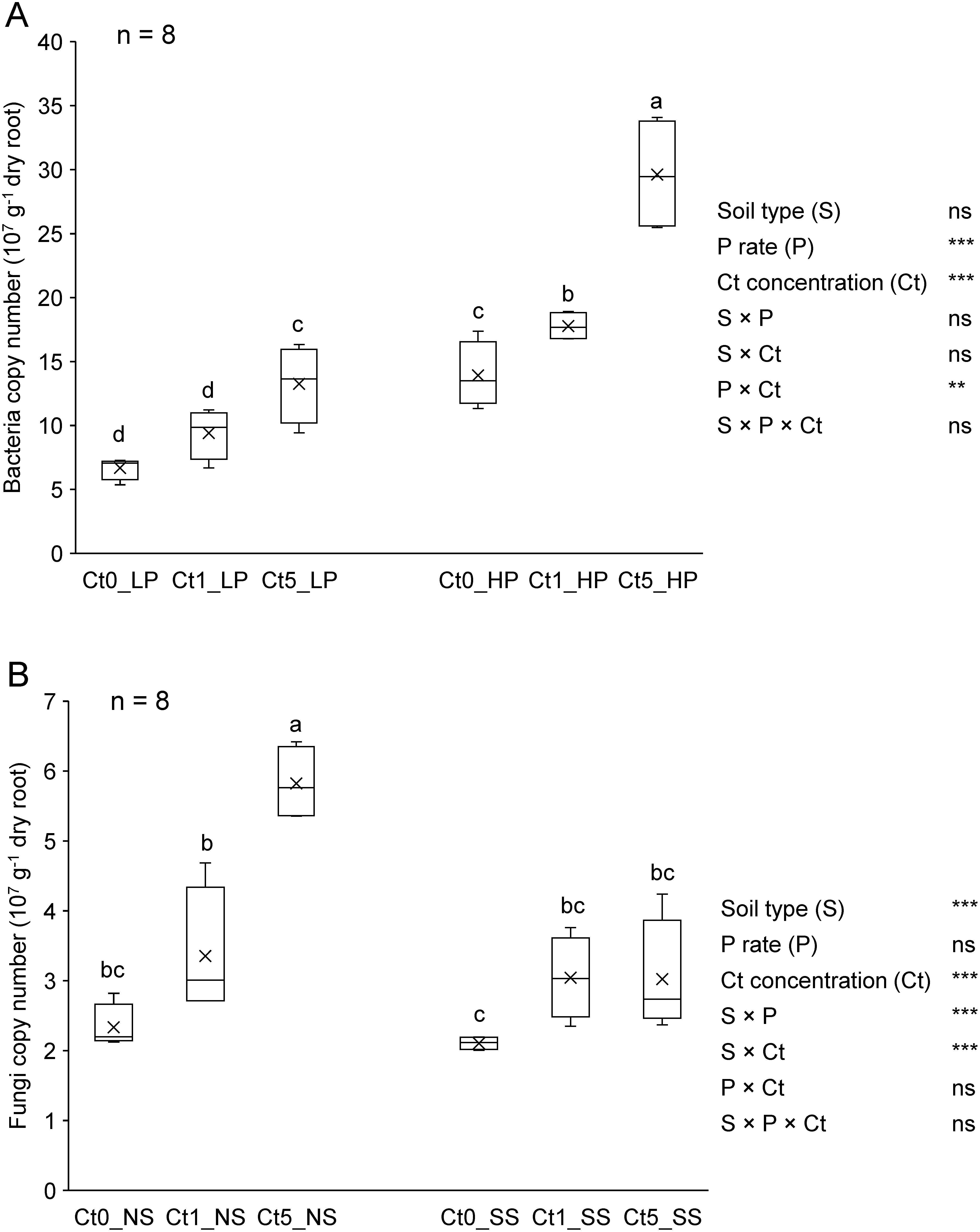
Figure 5. Abundance in komatsuna roots (at 32 dpi) of bacteria (A) as affected by inoculated Ct concentration and P application rate, and fungi (B) as affected by Ct concentration and soil type. For each variable, different letters indicate significant mean differences at *p*<0.05 in Tukey’s HSD test. In the ANOVA summaries, ** *p*<0.01, *** *p*<0.001, ns: not significant. Soil type refers to sterilized soil (SS) and non-sterilized soil (NS). P rates are 20 mg P kg^−1^ dry soil (Low P=LP) and 80 mg P kg^−1^ dry soil (High P=HP). Ct0, Ct1, and Ct5 are Ct concentrations at 0, 1, and 5% mass ratio of viable cells, dpi: days post-inoculation. *n* (top left corners)=number of biological replicates (5 replicates from SS+5 replicates from NS for A, 5 replicates from LP+5 replicates from HP for B).

Two-way ANOVA applied to root microbiome revealed no interaction effect on bacterial abundance between soil type and the P application rate, or between soil type and the Ct inoculation level. However, a significant P×Ct interaction was observed. Therefore, bacterial abundance is reportedly influenced by both the P application rate and Ct concentration (Figure 5A). Under Low P conditions, only Ct5 significantly increased the bacterial abundance in the roots, whereas under High P conditions, the bacterial abundance increased in a dose-dependent manner. In both P application treatments, Ct5 doubled the bacterial abundance in the roots. Although the soil type did not affect bacterial abundance, bacterial colonization of roots was significantly higher in the High P soil than in the Low P soil across all the Ct inoculation concentrations. In terms of root fungi, the P×Ct interaction was not significant, but positive interactions were observed between the soil type and P application rate, or between the soil type and Ct concentration. As a result, fungal abundance was influenced by the soil type and Ct inoculation concentration (Figure 5B). A higher fungal abundance was observed in komatsuna roots grown in non-sterilized soil with Ct5. No differences were observed between the effects of the three Ct concentrations on root fungal abundance under in the sterilized soil.

In stems, bacterial abundance also increased with increasing Ct inoculation concentration, with Ct5 being the most effective (Supplementary Figure S7A). However, under High P conditions, the bacterial abundance in stems was similar between the 5% and 1% Ct treatments. Overall, the bacterial abundance was higher in the High P soil. The fungal abundance in stems was influenced by the soil type, the phosphate application, and the Ct concentration (Supplementary Figure S7B). In both cases, fungal abundance increased with increasing Ct inoculation concentration. Furthermore, with the exception of Ct5, fungal abundance was higher in non-sterilized soil than in sterilized soil.

The abundance of bacteria in the leaves is shown in Supplementary Figure S8A. Under Low P conditions, the bacterial abundance was similar between Ct1 and Ct5, both of which were significantly higher than that under Ct0. In contrast, under High P conditions, Ct5 resulted in significantly higher bacterial abundance than both Ct1 and Ct0. However, at each Ct level, the bacterial abundance remained consistent between the Low and High P conditions. In contrast, fungal abundance in leaves followed a different trend (Supplementary Figure S8B). In non-sterilized soil, only the Ct5 increased fungal abundance in leaves, regardless of the P application rate. In sterilized soil, there were no significant differences in fungal abundance between the Ct0 and Ct1, while Ct5 showed a significantly higher abundance than Ct0 and Ct1.

## Discussion

In this study, we assessed the impact of *Colletotrichum tofieldiae* (Ct) inoculation on komatsuna growth, microbial community dynamics in rhizosphere soil and plant tissues, and P uptake under Low P and High P application rates of 20 mg kg^−1^ and 80 mg kg^−1^ dry soil, respectively. Our results revealed significant effects of Ct inoculation on plant growth and soil microbial abundance, highlighting its potential as a biological agent to increase nutrient uptake, particularly phosphorus, in leafy vegetable crops.

Previous studies, such as Hiruma et al. (2016), have shown that strains of Ct can invade the root tissues of *Arabidopsis thaliana*, in phosphorus-limited environments, and under sufficient P. Our findings align with these observations, further suggesting that Ct can colonize both the root and stem tissues of komatsuna and is more abundant in the rhizosphere, especially at the higher inoculation concentration of the 5% mass ratio. The effect was observed under both the Low P and High P application conditions. Proliferation of Ct in the soil and subsequent ability to colonize plant tissues, which may have contributed to the observed increase in total fungi in roots following inoculation, was associated with greater effects of Ct inoculation on plant biomass and P uptake, particularly in Low P conditions. Notably, plants grown under Low P with Ct5 treatments presented P uptake comparable to those grown under High P without inoculation, highlighting the potential of Ct to reduce fertilizer application while maintaining productivity.

The role of Ct in improving nutrient uptake may involve interactions with other soil microbes. Beneficial microorganisms, including mycorrhizal fungi, are well known to enhance nutrient uptake in nutrient-poor soils (Smith and Read 2008; van der Heijden et al. 2015). Interestingly, Hiruma et al. (2016) reported that Ct shares functional similarities with mycorrhizal fungi in enhancing P acquisition, suggesting that Ct may fulfill a comparable ecological role in *Brassicaceae* plants, which typically lack mycorrhizal associations. In our study, Ct inoculation led to an increase in total microbial abundance, including P-cycling microbes, in the rhizosphere. This supports the hypothesis that Ct may foster a synergistic interaction with soil microbiota, enhancing phosphorus mobilization and plant growth, as reflected in the higher P uptake. While Gonçalves et al. (2021) demonstrated that Ct can colonize the roots of maize and tomato in vitro, our study is the first to report the impact of Ct on the surrounding soil microbial communities, providing new insights into its potential to influence nutrient dynamics in komatsuna under natural soil conditions.

This shift in microbial community dynamics, particularly in the rhizosphere, likely contributes to the observed Ct effects in komatsuna growth, which were evident under both Low P and High P conditions. Interestingly, although the High P application rate (80 mg P kg^−1^) may not be considered excessive, our results suggest that Ct inoculation can still enhance nutrient uptake and growth, regardless of the soil’s P phosphorus status. Moreover, a high P application rate may not necessarily translate to increased available P (Shen et al. 2011), which can activate soil microbes to mobilize more accessible P (Richardson and Simpson 2011). In the present study, the significantly increased bacterial colonization of roots under the High P soil compared to the Low P soil across all Ct inoculation concentrations contrasts with Kou et al. (2023), who reported that increased phosphorus application does not alter bacterial abundance in soil. However, their findings may be valid within the specific context of their study parameters and may not be universally applicable across different soil types, environmental conditions, and fertilization practices. For instance, Wu et al. (2022) reported a global positive effect of phosphorus addition on soil microbial communities, with responses varying according to addition rate, mean annual temperature and precipitation, forms of phosphorus applied, and ecosystem type. An increase in the soil bacterial population following phosphorus application could potentially be associated with a subsequent increase in bacterial colonization of plant tissues. Interestingly, Ct was also detected in the non-inoculated soils, with a higher abundance under High P conditions. Such observations suggest that the soil may naturally harbor some Ct-like fungi, which can proliferate when additional phosphorus is available, partly explaining microbial shifts even in the absence of intentional inoculation. Our findings indicate that Ct inoculation likely creates conditions supporting the growth of a variety of soil microbes, including bacteria, fungi, and phosphorus-cycling microbes. The increased abundance of Ct in the rhizosphere, particularly at relatively high inoculation rates, implies that Ct actively shapes soil microbial communities to facilitate nutrient uptake and plant growth. In a similar study, Liu et al. (2021) reported an increase in wheat-associated microbiomes, particularly an enrichment of a dominant bacterium, *Stenophomonas rhizophila*, following inoculation with the fungus *Fusarium pseudograminearum*. This increase was associated with plant growth and induced disease resistance in the presence of pathogens. Although their ecological roles differ, with *F. pseudograminearum* acting primarily as a soil-borne pathogen and *Colletotrichum* species typically cause anthracnose (Guo et al. 2022; Liu et al. 2018) but sometimes forming mutualistic relationships, both fungi can influence plant-associated microbiomes. Furthermore, while *F. pseudograminearum* often disrupts plant root growth and nutrient acquisition, *Colletotrichum* may enhance plant nutrient uptake, including phosphorus acquisition. Despite belonging to different taxonomic families, they may share genetic pathways related to stress responses and nutrient metabolism. The synergistic relationship between Ct and other microbes has likely increased nutrient cycling in our study, enhancing komatsuna plant development. Collectively, these observations suggest that, like *F. pseudograminearum*, *Colletotrichum* may influence plant nutrient interactions, with our study providing initial evidence of Ct’s positive role in phosphorus acquisition in komatsuna.

An additional potential mechanism for the observed improvements in plant growth is the increased root surface area and nutrient absorption capacity resulting from Ct inoculation. Consistent with this, the observed increase in total biomass was accompanied by a proportional increase of both root and shoot biomass (separated data not shown). Plant growth-promoting microorganisms have been shown to increase root areas, thereby expending the ability of plants to absorb more nutrients from soil (Romano et al. 2020). Furthermore, photosynthates produced by aboveground tissues are transported via the phloem into the soil as carbohydrates, supporting microbial growth, including phosphorus-cycling microbes (Rengel and Marschner 2005; Trivedi et al. 2020). Enhanced microbial activity in turn likely contributes to greater nutrient availability in the rhizosphere. Consequently, plant root growth was enhanced by Ct inoculation, and shoot growth parameters, including leaf length and leaf number, were significantly increased in the Ct-inoculated plants, potentially due to a combination of Ct-mediated root growth promotion and Ct-hyphae-mediated P uptake.

The highest inoculation level (Ct5) consistently outperformed the lower (Ct1) and non-inoculated (Ct0) treatments in terms of plant growth, suggesting a dose-dependent response. Specifically, plants treated with Ct5 exhibited significantly greater increases in root and shoot biomass (overall plant biomass shown), leaf length, and leaf number compared to those in the lower inoculation groups. especially under Low P soil conditions. No difference was found between Ct5 and Ct1, and only Ct5 increased plant growth compared to Ct0 in the High P soil conditions. These observations indicate that, within the tested inoculum range, increasing the dose enhances the beneficial effects of *Colletotrichum* on plant growth. However, while our study demonstrates that relatively high inoculation rates are beneficial, it remains unclear whether further increasing the Ct dose beyond 5% would continue to enhance plant growth or if there is a threshold beyond which the effect levels off or becomes detrimental. Further research is needed to explore the optimal range of Ct inoculation, as well as to investigate potential trade-offs or diminishing returns with higher doses, including any potential negative effects on the plant or microbial community dynamics.

The sterilized and non-sterilized soil treatments provided additional insights into the role of indigenous soil microorganisms in the effectiveness of Ct inoculation. Ct inoculation influenced most parameters similarly in both sterilized and non-sterilized soils, suggesting minimal competition with native microbes and reinforcing its potential as a biofertilizer for leafy vegetables. In non-sterilized soil, microbial communities, that are already adapted to the environment contribute to the effectiveness of bioinoculants in nutrient cycling (Samantaray et al. 2024). Our study observed that Ct interacted positively with these native communities, as evidenced by a significant increase in the abundance of microbes, including those involved phosphorus-cycling. Such findings suggest that Ct may support and possibly enhance microbial activity, potentially contributing to more efficient nutrient cycling in the soil. In sterilized soil, where native microbes are absent, Ct is predicted to have a more direct effect on newly introduced microbial populations, likely because of its initial colonization of the previously sterilized environment. Although differences were observed in a few parameters, such as fungi in the roots, these differences were limited irrespective of soil sterilization. Overall, Ct remained effective under both sterilized and natural soil conditions, highlighting the potential of using biological amendments, such as Ct, to increase P uptake in both nutrient-poor and nutrient-rich soils. These findings suggest that Ct inoculation could contribute to more sustainable nutrient management practices, potentially benefiting eco-friendly agricultural systems.

While this study provides valuable insights into the role of Ct in improving P uptake and plant growth, further research is needed to assess the long-term effects of Ct inoculation under field conditions, particularly in P-limited environments. Future studies could also explore interactions between Ct and other microorganisms in the soil and how these interactions influence nutrient cycling and plant growth over time.

## Conclusion

This study demonstrated the potential of *Colletotrichum tofieldiae* (Ct) inoculation to enhance phosphorus uptake and komatsuna growth under natural soil conditions, in both P-deficient and P-sufficient soils. Ct colonized komatsuna plant tissues from soils, improving nutrient absorption and altering microbial community dynamics in the rhizosphere and plant tissues. The increased abundance of phosphorus-cycling microbes suggests that Ct works synergistically with other soil microorganisms to promote nutrient cycling and growth. The effects of Ct were consistent across varying applied phosphate amount and soil sterilization conditions, emphasizing its versatility as a biofertilizer for crops in nutrient-poor soils. The ability of Ct to increase nutrient availability and reduce the need for chemical fertilizers underscores its potential for promoting sustainable agricultural practices.

## Description of supplementary files

Supplementary Figure S1. Determination of the applied P rates for the main experiment.

Supplementary Figure S2. Total biomass of komatsuna (at 32 dpi) as affected by inoculated Ct concentration and soil type.

Supplementary Figure S3. Leaf count and leaf length of komatsuna during growth as affected by inoculated Ct concentration and P application rate.

Supplementary Figure S4. Abundance of microbes (bacteria+fungi) in komatsuna rhizosphere soil (at 32 dpi) as affected by inoculated Ct concentration and soil type.

Supplementary Figure S5. Ct abundance in komatsuna rhizosphere soil (at 32 dpi) as affected by inoculated Ct concentration and soil type.

Supplementary Figure S6. Abundance of selected phosphorus-cycling microbial genes (*gcd*, *pqqE*, *phoD*) in komatsuna rhizosphere soil (at 32 dpi) as affected by inoculated Ct concentration and soil type.

Supplementary Figure S7. Abundance in komatsuna stems (at 32 dpi) of bacteria as affected by inoculated Ct concentration and P application rate, and fungi as affected by Ct concentration and soil type.

Supplementary Figure S8. Abundance in komatsuna leaves (at 32 dpi) of bacteria as affected by inoculated Ct concentration and P application rate, and fungi as affected by Ct and soil type.

Supplementary Table S1. Primer sequences of the amplified microbial genes.
