# Supplementary material for: Colletotrichum tofieldiae enhances phosphorus uptake and biomass production and alters the microbial interactions in the rhizosphere of komatsuna (Brassica rapa var. perviridis) grown in phosphorus-deficient farm soils
Source: Plant Biotechnol (Tokyo). 2025 Sep 25;42(3):371–82. doi: 10.5511/plantbiotechnology.25.0529a (PMC12573603; doi:10.5511/plantbiotechnology.25.0529a)
Supplement: Supplementary Data [file plantbiotechnology-42-3-25.0529a_s001.pdf]

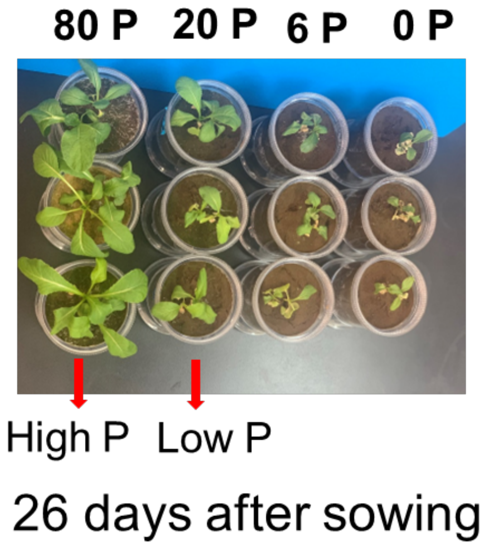

Supplementary Figure S1. Determination of the applied P rates for the main experiment
0 P, 6 P, 20 P, 80 P are 0, 6, 20, 80 mg P kg^{-1} dry soil, respectively. Based on growth pattern, 20 P and 80 P are used to represent the Low P and High P, respectively.

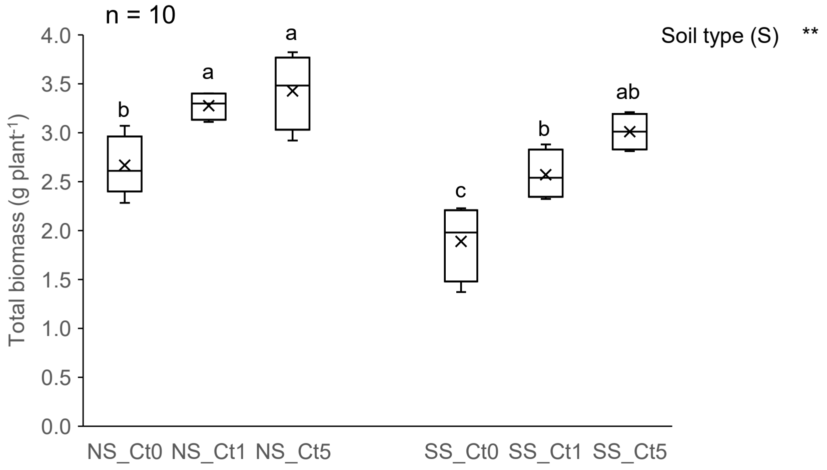

Supplementary Figure S2. Total biomass of komatsuna (at 32 dpi) as affected by inoculated Ct concentration and soil type. Different letters indicate significant mean differences at $p < 0.01$. Soil type refers to sterilized soil (SS) and non-sterilized soil (NS). P rates are 20 mg P kg^{-1} dry soil (Low P = LP) and 80 mg P kg^{-1} dry soil (High P = HP). Ct0, Ct1, and Ct5 are Ct concentrations at 0%, 1%, and 5% mass ratio of viable cells, dpi: days post-inoculation. n (top left corner) = number of biological replicates (5 replicates from LP + 5 replicates from HP).

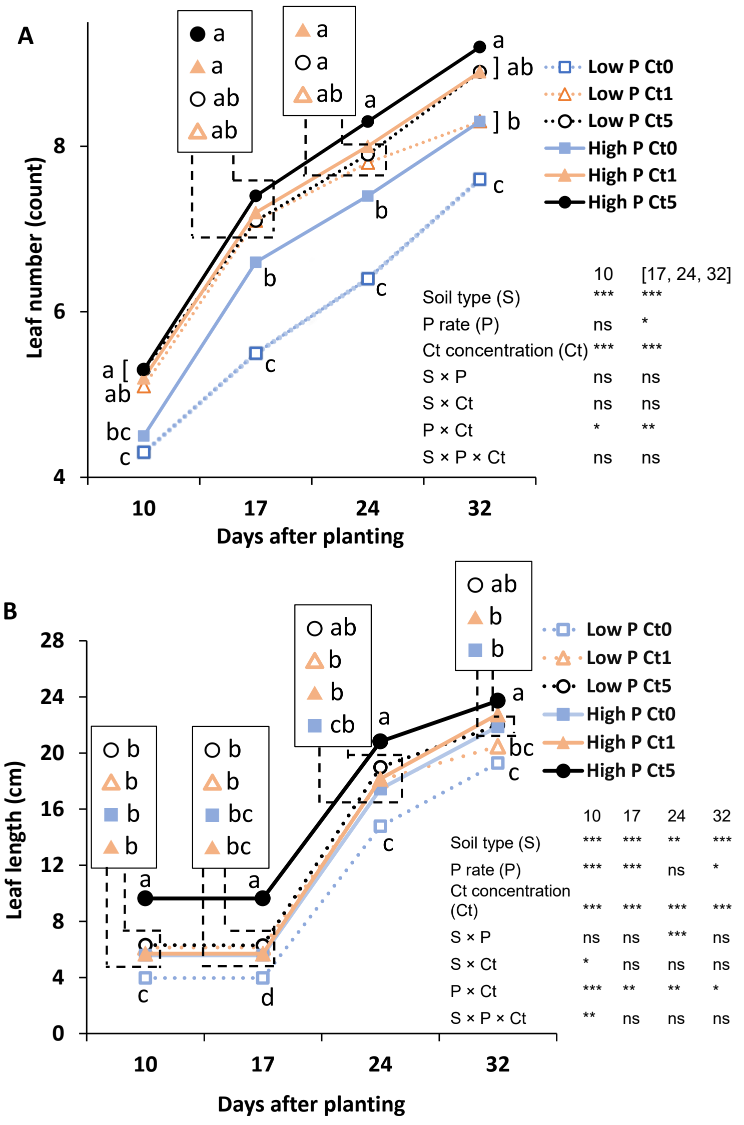

Supplementary Figure S3. Leaf count (A) and leaf length (B) of komatsuna during growth as affected by inoculated Ct concentration and P application rate. At each measuring date, different letters indicate significant mean differences at $p < 0.05$ in Tukey's HSD test. In the ANOVA summary, $*p < 0.05$, $**p < 0.01$, $***p < 0.001$, ns. not significant. Soil type refers to sterilized soil and non-sterilized soil. P rates are 20 mg P kg^{-1} dry soil (low P) and 80 mg P kg^{-1} dry soil (high P). Ct0, Ct1, and Ct5 are Ct concentrations at 0%, 1%, and 5% mass ratio of viable cells, dpi: days post-inoculation.

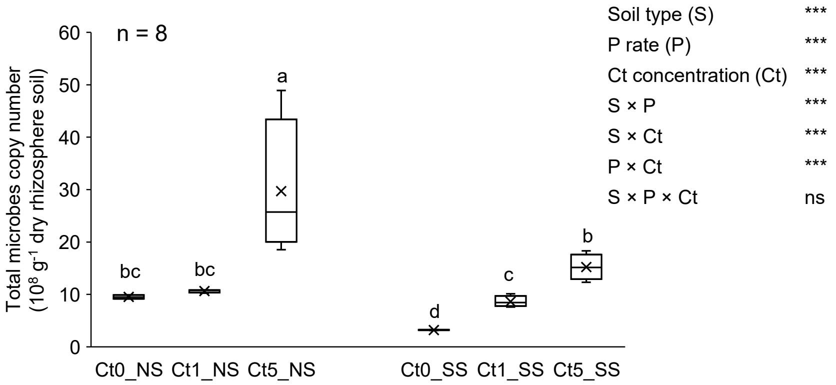

Supplementary Figure S4. Abundance of microbes (bacteria + fungi) in komatsuna rhizosphere soil (at 32 dpi) as affected by inoculated Ct concentration and soil type. Different letters indicate significant mean differences at $p < 0.05$ in Tukey's HSD test. In the ANOVA summary, $***p < 0.001$, ns. not significant. Soil type refers to sterilized soil (SS) and non-sterilized soil (NS). P rates are 20 mg P kg^{-1} dry soil (Low P) and 80 mg P kg^{-1} dry soil (High P). Ct0, Ct1, and Ct5 are Ct concentrations at 0%, 1%, and 5% mass ratio of viable cells, dpi: days post-inoculation. n (top left corner) = number of biological replicates (5 replicates from LP + 5 replicates from HP).

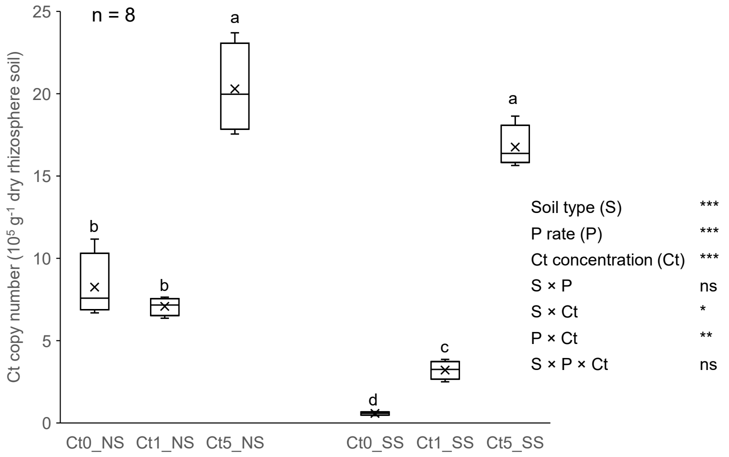

Supplementary Figure S5. Ct abundance in komatsuna rhizosphere soil (at 32 dpi) as affected by inoculated Ct concentration and soil type. Different letters indicate significant mean differences at $p < 0.05$ in Tukey's HSD test. In the ANOVA summary, *$p < 0.05$, **$p < 0.01$, ***$p < 0.001$, ns. not significant. Soil type refers to sterilized soil (SS) and non-sterilized soil (NS). P rates are 20 mg P kg^{-1} dry soil (Low P) and 80 mg P kg^{-1} dry soil (High P). Ct0, Ct1, and Ct5 are Ct concentrations at 0%, 1%, and 5% mass ratio of viable cells, dpi: days post-inoculation. n (top left corner) = number of biological replicates (5 replicates from LP + 5 replicates from HP).

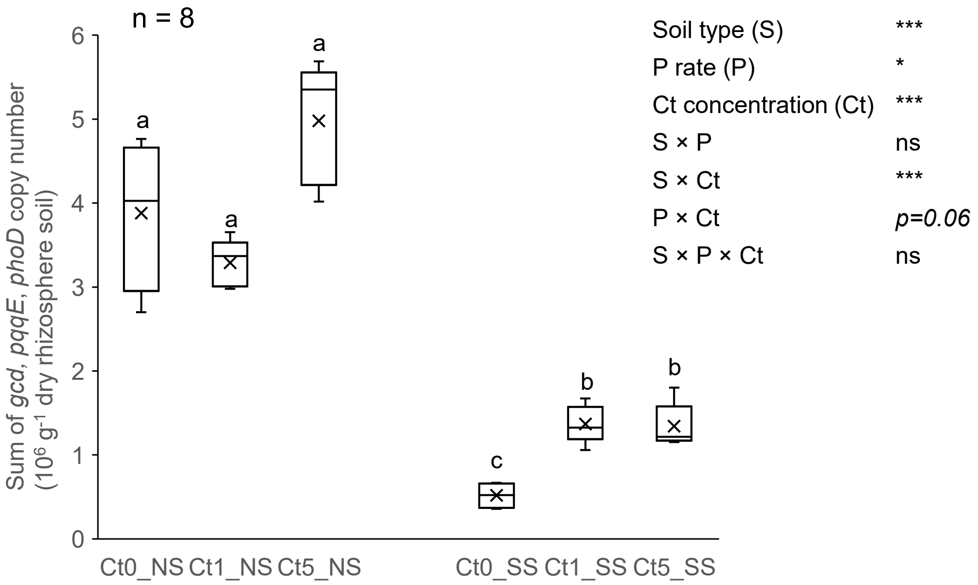

Supplementary Figure S6. Abundance of selected phosphorus-cycling microbial genes (*gcd*, *pqqE*, *phoD*) in komatsuna rhizosphere soil (at 32 dpi) as affected by inoculated Ct concentration and soil type. Different letters indicate significant mean differences at $p < 0.05$ in Tukey's HSD test. In the ANOVA summary, $*p < 0.05$, $***p < 0.001$, ns. not significant. Soil type refers to sterilized soil (SS) and non-sterilized soil (NS). P rates are 20 mg P kg^{-1} dry soil (Low P) and 80 mg P kg^{-1} dry soil (High P). Ct0, Ct1, and Ct5 are Ct concentrations at 0%, 1%, and 5% mass ratio of viable cells, dpi: days post-inoculation. n (top left corner) = number of biological replicates (5 replicates from LP + 5 replicates from HP).

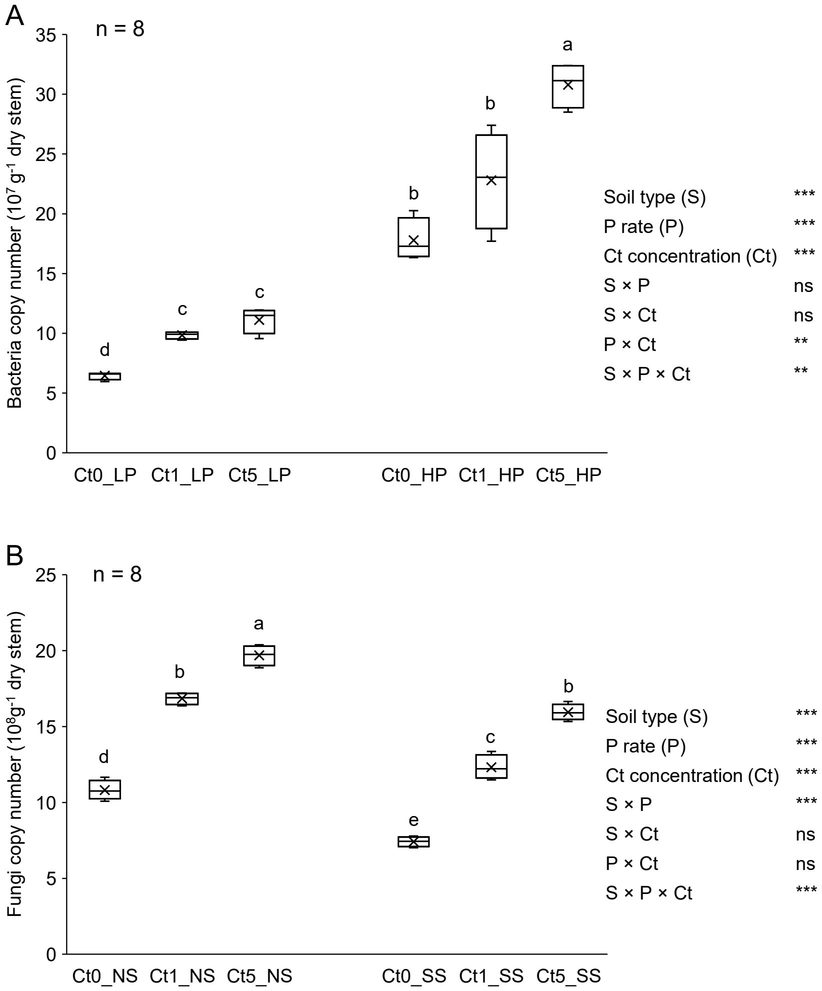

Supplementary Figure S7. Abundance in komatsuna stems (at 32 dpi) of bacteria (A) as affected by inoculated Ct concentration and P application rate, and fungi (B) as affected by Ct concentration and soil type. For each variable, different letters indicate significant mean differences at $p < 0.05$ in Tukey's HSD test. In the ANOVA summary, $^{**}p < 0.01$, $^{***}p < 0.001$, ns. not significant. Soil type refers to sterilized soil (SS) and non-sterilized soil (NS). P rates are 20 mg P kg^{-1} dry soil (Low P = LP) and 80 mg P kg^{-1} dry soil (High P = HP). Ct0, Ct1, and Ct5 are Ct concentrations at 0%, 1%, and 5% mass ratio of viable cells, dpi: days post-inoculation. n (top left corners) = number of biological replicates (5 replicates from SS + 5 replicates from NS for A, 5 replicates from LP + 5 replicates from HP for B).

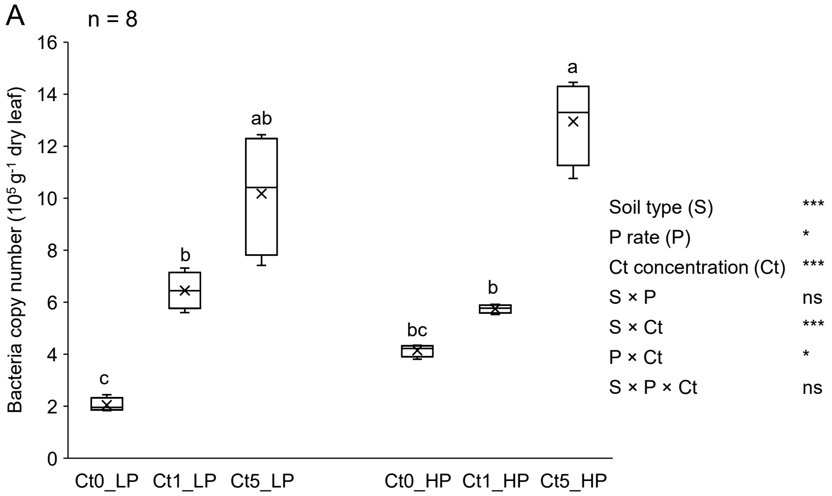

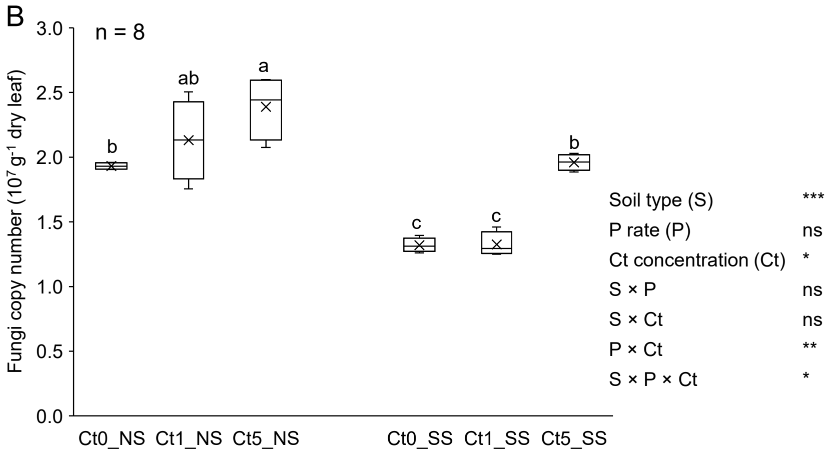

Supplementary Figure S8. Abundance in komatsuna leaves (at 32 dpi) of bacteria (A) as affected by inoculated Ct concentration and P application rate, and fungi (B) as affected by Ct and soil type. For each variable, different letters indicate significant means differences at $p < 0.05$ in Tukey's HSD test. In the ANOVA summaries, *$p < 0.05$, **$p < 0.01$, ***$p < 0.001$, ns. not significant. Soil type refers to sterilized soil (SS) and non-sterilized soil (NS). P rates are 20 mg P kg^{-1} dry soil (Low P = LP) and 80 mg P kg^{-1} dry soil (High P = HP). Ct0, Ct1, and Ct5 are Ct concentrations at 0%, 1%, and 5% mass ratio of viable cells, dpi: days post-inoculation. n (top left corners) = number of biological replicates (5 replicates from SS + 5 replicates from NS for A, 5 replicates from LP + 5 replicates from HP for B).

Supplementary Table S1: Primer sequences of the amplified microbial genes

Genes	Primers	Sequences	References	Ta (qPCR)
16S rRNA	Bac8Fmod	AGAGTTTGATYMTGGCTCAG	Loy et al., 2002	60ºC
	Bac338Rabc	GCWGCCWCCCGTAGGWGT	Nercessian et al. 2005	
ITS	ITS1	TCCGTAGGTGAACCTGCGG	White et al., 1990	60ºC
	ITS2	GCTGCGTTCTTCATCGATGC		
CT04_11973	Cttub-F	AGTCTTTCCTGATCCCGACC	Hiruma et al., 2016	57ºC
	Cttub-R	AAGTGGCCAGATCAAGTCAA		
gcd	gcd-F	GACCTGTGGGACATGGACGT	Chen et al., 2016	61ºC
	gcd-R	GTCCTTGCCGGTGTAGSTCATC		
pqqE	pqqE-F	TCCGTGGCTATGAGTGGA	An and Moe, 2016	61ºC
	pqqE-R	CATCACCGGTCAGCATGAA		
phoD	phoDF733	TGGGAYGATCAYGARGT	Ragot et al., 2015	59ºC
	phoDR1083	CTGSGCSAKSACRTTCCA		

Ta: Annealing temperature
CT04_11973: Ct tubulin 2 sequence

An R and Moe L (2016) Regulation of Pyrroloquinoline Quinone-Dependent Glucose Dehydrogenase Activity in the Model Rhizosphere-Dwelling Bacterium *Pseudomonas putida* KT2440. *Appl Environ Microbiol* 82(16): 4955–64. doi: 10.1128/AEM.00813-16

Chen W, Yang F, Zhang L, Wang J (2016) Organic acid secretion and phosphate solubilizing efficiency of *pseudomonas* sp. PSB12: Effects of phosphorus forms and carbon sources. *Geomicrobiol J* 33: 870–877. https://doi.org/10.1080/01490451.2015.1123329 (2016)

Hiruma K, Gerlach N, Sacristán S, Nakano RT, Hacquard S, Kracher B et al. (2016) Root endophyte *Colletotrichum tofieldiae* confers plant fitness benefits that are phosphate status dependent. *Cell* 165: 464–474

Loy A, Lehner A, Lee N, Adamczyk J, Meier H, Ernst J, Schleifer KH, Wagner M (2002) Oligonucleotide microarray for 16S rRNA gene-based detection of all recognized lineages of sulfate-reducing prokaryotes in the environment. *Appl Environ Microbiol* 68: 5064–5081. https://doi.org/10.1128/AEM.68.10.5064-5081.2002

Nercessian O, Fouquet Y, Pierre C, Prieur D, Jeanthon C (2005) Diversity of bacteria and archaea associated with a carbonate-rich metalliferous sediment sample from the rainbow vent field on the Mid-Atlantic Ridge. *Environ Microbiol* 7: 698–714. doi: 10.1111/j.1462-2920.2005.00744.x

Ragot SA, Kertesz MA, Bunemann EK (2015) *phoD* Alkaline Phosphatase Gene Diversity in Soil. *Appl Environ Microbiol* 81(20): 7281–9. doi: 10.1128/AEM.01823-15

White TJ, Bruns TF, Lee SB, Taylor JW (1990) Amplification and direct sequencing of fungal ribosomal RNA genes for phylogenetics. In: Innis MA, Gelfand DH, Sninsky JJ, White TJ (eds) *PCR Protocols: A Guide to Methods and Applications*. Academic Press, San Diego, CA, pp 315–322
